# Human Arm Workout Classification by Arm Sleeve Device Based on Machine Learning Algorithms

**DOI:** 10.3390/s23063106

**Published:** 2023-03-14

**Authors:** Sehwan Chun, Sangun Kim, Jooyong Kim

**Affiliations:** 1 Department of Organic Materials and Fiber Engineering, Soongsil University, Seoul 156-743, Republic of Korea; 2 Department of Smart Wearable Engineering, Soongsil University, Seoul 156-743, Republic of Korea

**Keywords:** EMG, arm workout classification, smart wearables, smart clothing, textile-based electrode, machine learning, decision tree, SVM, KNN

## Abstract

Wearables have been applied in the field of fitness in recent years to monitor human muscles by recording electromyographic (EMG) signals. Understanding muscle activation during exercise routines allows strength athletes to achieve the best results. Hydrogels, which are widely used as wet electrodes in the fitness field, are not an option for wearable devices due to their characteristics of being disposable and skin-adhesion. Therefore, a lot of research has been conducted on the development of dry electrodes that can replace hydrogels. In this study, to make it wearable, neoprene was impregnated with high-purity SWCNTs to develop a dry electrode with less noise than hydrogel. Due to the impact of COVID-19, the demand for workouts to improve muscle strength, such as home gyms and personal trainers (PT), has increased. Although there are many studies related to aerobic exercise, there is a lack of wearable devices that can assist in improving muscle strength. This pilot study proposed the development of a wearable device in the form of an arm sleeve that can monitor muscle activity by recording EMG signals of the arm using nine textile-based sensors. In addition, some machine learning models were used to classify three arm target movements such as wrist curl, biceps curl, and dumbbell kickback from the EMG signals recorded by fiber-based sensors. The results obtained show that the EMG signal recorded by the proposed electrode contains less noise compared to that collected by the wet electrode. This was also evidenced by the high accuracy of the classification model used to classify the three arms workouts. This work classification device is an essential step towards wearable devices that can replace next-generation PT.

## 1. Introduction

**Figure 1:**
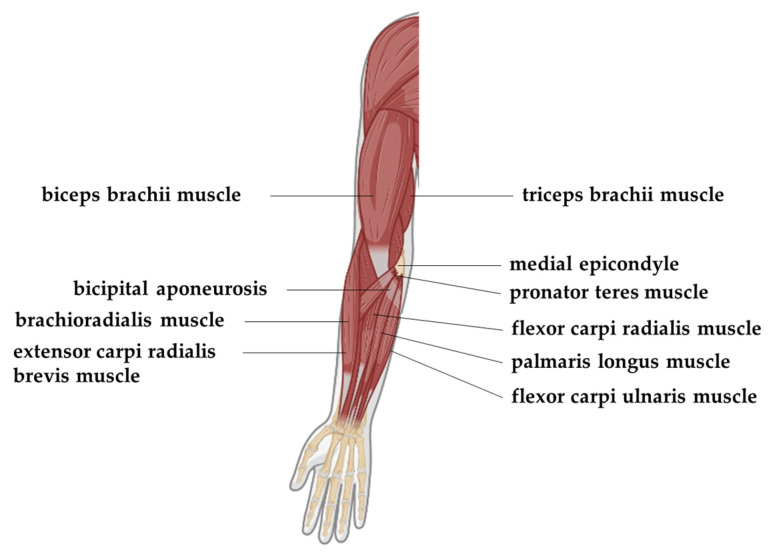
Anatomy of the human forearm (anterior view, superficial layer).

Resistance training—which is sometimes called weight training or strength training—has been considered to be one of the most fundamental exercises to improve muscular strength and endurance [1]. When the COVID-19 pandemic broke out, people began to care about their health more than ever. Social distancing made home-based exercising a trend. In fact, resistance training, with the benefits of improvement in both physical and mental health, and the advantage of being able to work out with or without equipment, has made inroads into people’s regular home-based workout routines. In arm resistance training, three of the most common workouts are Wrist Curl, Biceps Curl, and Dumbbell Kickback. Three muscles that get involved in these workouts are triceps brachii, biceps brachii, and brachioradialis, respectively. The positions of these muscles are illustrated in Figure 1, in which triceps brachii functions as the major extension of the forearm, biceps brachii is responsible for the flexion and supination of the forearm, and brachioradialis serves as the weak flexor of the forearm [2].

In the past, hydrogel (Ag/AgCl) electrodes were used mostly to monitor muscle activity. However, some recent studies have proposed several methods to monitor EMG signals, in which they replaced them with dry electrodes because of their superiority over the other type in terms of the manufacturing procedure, users’ experience, and measurement performance [3,4,5,6,7,8,9,10]. In [9], The EMG signal was analyzed by varying the embroidery pattern of the conductive yarn. As the stretchable area increased, the shape deformation was more stable. In addition, as the area in contact with the skin increased, the impedance decreased due to more contact. A dry electrode capable of generating a morphologically stable signal was fabricated. In [10], a dry electrode was produced by printing silver paste on PET material with a round shape. Its performance was evaluated by obtaining arm muscle data in the form of an armband and comparing it with the conventional wet electrode; the EMG signal was measured by various types of electrode conductive lines and waves. In addition, past studies have classified aerobic exercises such as cycling, running, and jumping in a wearable form, and have focused on motion classification such as finger motion recognition using dry electrodes [11,12,13,14,15]. In [13], the hydrogel electrode is replaced with a fabric electrode and seven finger movements are classified as EMG signals.

One of the most common applications of EMG signals is to detect human motions, which is mainly based on the high variability of its amplitude during specific gestures or motions. In particular, during muscle contractions, EMG provides a considerable amount of information [16,17,18,19]. In fitness training, monitoring muscle activity can be applied to detect the workout, to additionally enhance the effectiveness of the training process [20,21]. Formenti, D et al. proposed a method of using infrared thermal images (IRTG) to detect muscle activation during resistance training. Both studies pointed out the correlation between skin surface temperature and muscle activation. Some advantages of IRTG including contact-free technology, low setup cost, and low image processing complexity have been recognized, although this approach still presented some drawbacks, such as the effects of perspiration and muscle fatigue being diminished [22]. Furthermore, there were differences in thermoregulatory response among athletic and untrained subjects [23], and healthy and injured athletes [24]. Moreover, this method is not suitable for monitoring muscle activity in real-time. Instead of exploring muscle activation based on the correlation between it and body temperature during training, the second approach focuses on monitoring neuromuscular activity during exercise [20]. In this study, a fitness shirt attached with eight EMG sensors was deployed to record EMG signals; however, the wearable function of this device is not practical because the type of electrode used in this study is a Ag/AgCl-based electrode which is not reusable. Therefore, there is a need for a device that can be manufactured in a wearable form using dry electrodes, able to collect and process data in real-time, that helps the users monitor their muscles during their fitness training.

In this study, the authors aimed to develop a wearable device to record EMG signals, in the form of an arm sleeve attached with nine textile-based electrodes. To verify the performance of this device, some machine learning classification models were applied to detect and classify three of the most typical arm-target workouts: Wrist Curl, Biceps Curl, and Dumbbell Kickback. The procedure of manufacturing the arm sleeve and the comparison of the performance of the textile-based sensor and the hydrogel-based sensor are described in detail in the Materials and Methods part. Potential developments of this device will also be discussed later in this paper.

## 2. Materials and Methods

### 2.1. Sensor Fabrication

**Figure 2:**
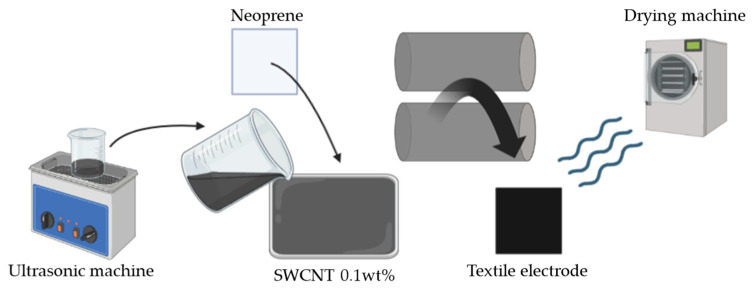
The fabrication process of the textile-based electrode.

The hydrogel has a disadvantage in that it needs to be replaced periodically because its performance is weakened by sweat or contaminants. However, neoprene has resistance to oil, high temperature, and water, and it has been used for a long time for industrial purposes and wetsuits [25]. In addition, there is an air gap, so it can sufficiently adhere to the skin by the pressure of clothing during a workout [26]. Therefore, in this research, neoprene was chosen as the material used for the textile-based electrode (KOLON Co., Ltd., Seoul, Republic of Korea) that contains polyester (PE) and spandex (SP) mixed with a ratio of 88:12, respectively, with the thickness of 1.05 mm and the density of 40 gauge. Single-Walled Carbon Nanotubes (SWCNT) (KH CHEMICALS Co., Ltd., Seoul, Republic of Korea) that contains SWCNT: 20~30 wt%/Transition metals: 15~30 wt%/Water soluble impurities: 45~60%, average diameter of 1.2 nm. It used as the material for conductivity. To fabricate the electrode, SWCNTs ink was stirred for at least 1 h in the Ultrasonic machine with a spin speed of 800 rpm; then, the compound liquid was put in an ultrasonic machine to avoid incorporating air bubbles to ensure uniform distribution on the fabric’s surface [27]. Neoprene was sufficiently immersed in 0.1wt% of water base SWCNT for 1 min, and conductive particles were penetrated through a dipping padding machine (DAELIM Lab., Seoul, Republic of Korea). After that, the two-way drying machine (DAELIM Lab., Seoul, Republic of Korea) was used to get rid of the excess water in the fabrics, with temperatures ranging from 80 °C to 100 °C, for 5 min, and the speed of the circulation fan was 1500 rpm. Finally, the fabric was maintained for 1 h under normal room temperature. The whole fabrication process is illustrated in Figure 2.

**Figure 3:**
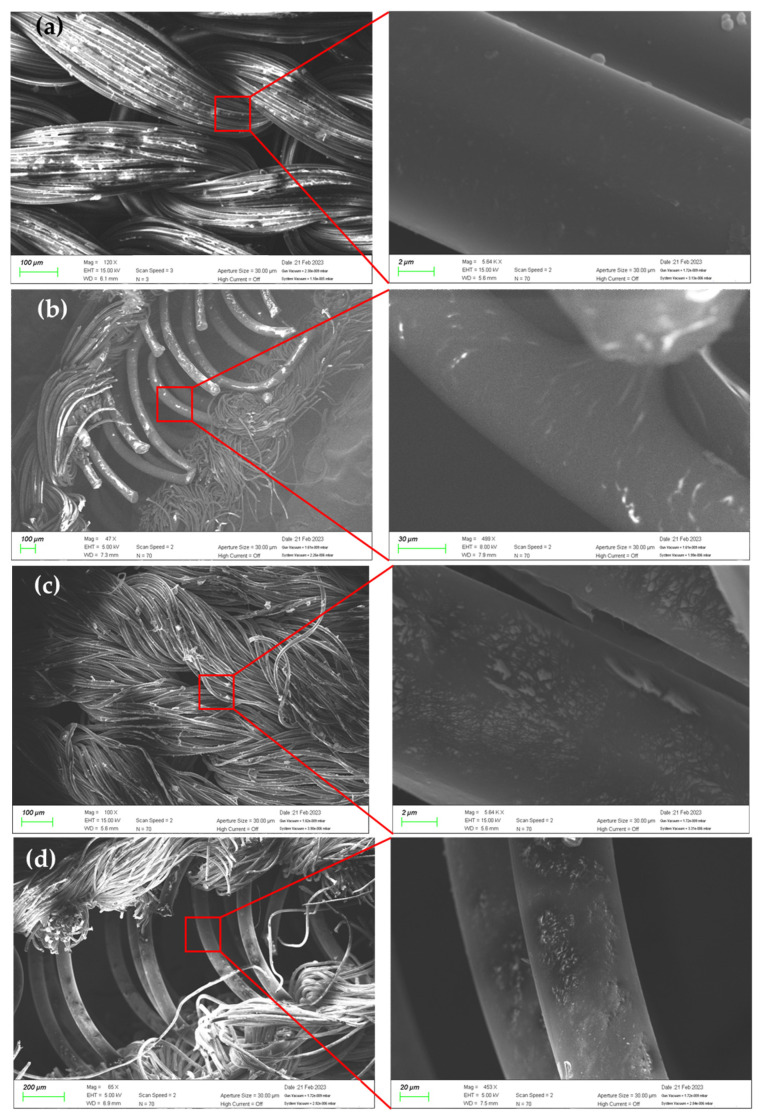
The fabrication process of the textile-based electrode (**a**) top of non-conductive neoprene, (**b**) cross-section of non-conductive neoprene, (**c**) top of conductive neoprene, (**d**) cross-section of conductive neoprene.

A scanning electron microscope (Gemini SEM 300 from ZEISS Co., Ltd., Oberkochen, Germany) was used to evaluate the structure and surface morphology of the SWCNT-coated neoprene. SEM microphotographs from 5 k to 15 k magnification of coating samples are shown in Figure 3. As can be seen, the CNTs were well dispersed around the surface of the filament yarn.

**Figure 4:**
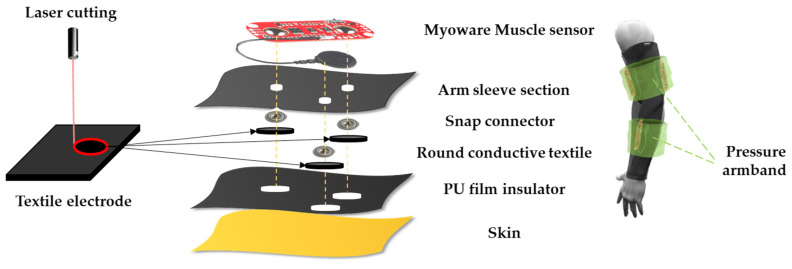
Arm sleeve device design.

The arm sleeve is made of PE/SP fabric with a polyester/spandex ratio of 88:12, respectively (Lightweight running sleeve, Nike, Portland, OR, USA), same material composition as Neoprene. It is durable with a double sewing system and suitable for a workout because of its capability to release sweat and moisture quickly. The arm sleeve device consists of a low-cost EMG circuit, which is widely available in the market under the name MyoWare [28], that needs three electrodes to record the EMG signal. The two which are placed along the muscle are for obtaining the potential difference, and the other one is the reference electrode, which is constant and serves as a reference when calculating the potential difference. These three electrodes were replaced with conductive textile-based electrodes (dry type) instead of hydrogel electrodes (wet type). To make the arm sleeve (Figure 4), conductive neoprene textile was cut into a circle shape by laser cutting with a diameter of 25 mm, which is the same as the dimension of the hydrogel-based electrode. First, holes were made in the arm sleeve for the snap connector to enter and be fixed. A circle-shaped conductive textile was placed under each snap connector and secured with a thin PU film. The PU film was also cut by laser with a diameter of 20 mm, and the area in contact with the skin was set to 314.16 ${mm}^{2}$ (π ×$r^{2}$) by covering the conductive textile with a diameter of 20 mm.

### 2.2. Data Acquisition Protocol

**Figure 5:**
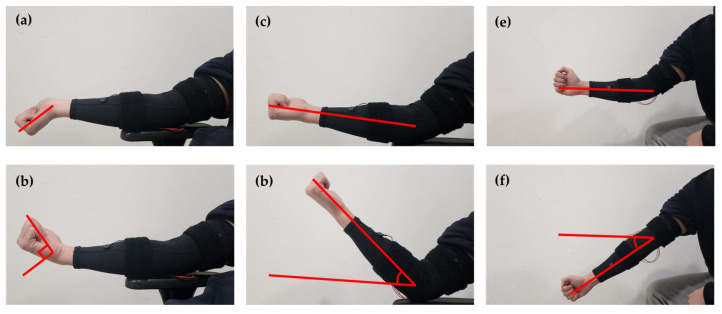
Perform the workout: (**a**) Wrist Curl (contraction), (**b**) Wrist Curl (extension), (**c**) Biceps Curl (contraction), (**d**) Biceps Curl (extension), (**e**) Dumbbell Kickback (extension), (**f**) Dumbbell Kickback (contraction).

In this study, the authors aimed to classify three typical arm workouts: Wrist Curl, Biceps Curl, and Dumbbell Kickback. The arm sleeve device equipped with the fabricated textile-based electrodes and the MyoWare board was used to record the EMG signal for the workout classification task. The sleeve was tightly fitted but still comfortable for each participant. During the test, four participants who had similar ages and heights (age: 28–29, height: 178–182, male, right-handed, Table 1) were asked to wear the arm sleeve device while performing the workouts. Specifically, the workout starts with 30 s of rest while data are collected for stabilization. Next, they were asked to carry out 10 repetitions of each workout, followed by 1 min rest. During the Wrist Curl workout, the participants fixed their forearms, then raised and lowered their right wrist within the range of −45 to +45 degrees based on the wrist orientation. During Biceps Curl and Dumbbell Kickback, the participants fixed their upper arms and raised and lowered their forearms within the range of 0 to 45 degrees based on their wrist orientation. The illustration of 3 workouts is shown in Figure 5.

**Figure 6:**
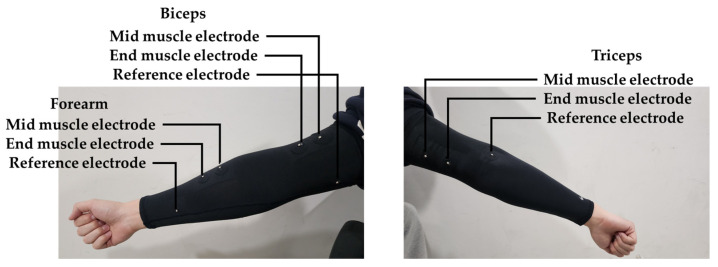
Electrode position of arm sleeve device.

Figure 6 shows the position of electrodes on the participant’s right arm. The electrodes were attached to the MyoWare boards and placed along the interested muscles, which are brachioradialis, biceps brachii, and triceps brachii [10,11,12]. Brachioradialis is mostly activated during Wrist Curl, while biceps brachii and triceps brachii can be activated by moving the forearm up and down when the upper arm is fixed [13,14] (Figure 5). EMG envelope signal was recorded by the SIG pin of the Myoware circuit, then collected by Arduino Nano 33 BLE (4.5~21 V, 64 MHz) and transmitted to the computer via Bluetooth Low Energy (BLE) with the sampling frequency of 20 Hz. Three alkaline batteries (1.5 V) were connected in series to serve as the power supplier for the Arduino board. Data were then mapped from analog value, ranging from 0 to 1023, for voltage ranging from 0 to 3.3 V.

Normalization means conceptually adjusting values measured on different scales to a common scale before averaging. Since each person’s EMG activity is different, normalization was performed. Normalization is calculated as follows:

(1) $$Normalization=\frac{A-A_{minimum}}{A_{maximum}-A_{minimum}}$$

Standard deviation (STD) is a measure of the difference between each measurement value and the mean and indicates the degree of spread of the data. A low STD indicates that the data points are close to the mean, and a high STD indicates that the data points are far from the mean. STD is calculated as follows:

(2) $$STD=\sqrt{\frac{\sum_{i=1}^{n}{\left|A_{i}-\overset{-}{A}\right|}^{2}}{n-1}}$$

Low-pass filter (LPF) is filter that attenuates signals with frequencies above a specific cut-off frequency and passes only signals with frequencies below the cut-off frequency. The cut-off frequency was set to 5 Hz to filter the frequency by dry electrode.

(3) $$f_{c}=\frac{1}{2\pi RC}$$

### 2.3. Data Pre-Processing

**Figure 7:**
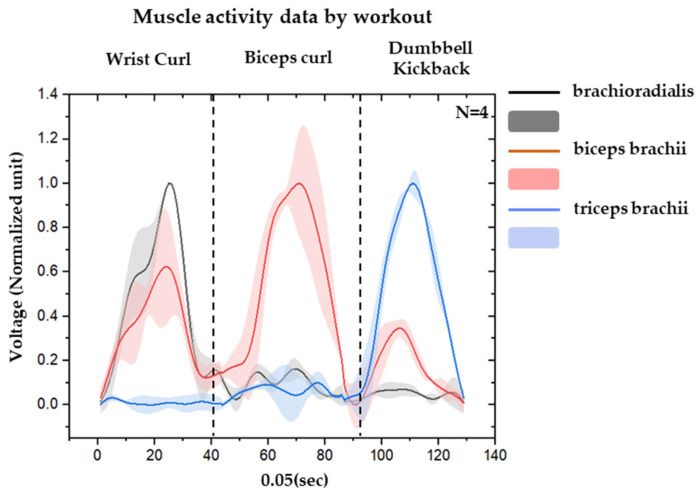
Rescaled EMG data and standard deviation of 4 participants.

The EMG envelope data, both recorded by the hydrogel-based and the textile-based sensor, which are illustrated in Figure 7, were loaded into MATLAB for further signal processing steps. Each channel corresponds to one muscle following this order: brachioradialis, biceps brachii, and triceps brachii. A low-pass filter with a cut-off frequency of 5 Hz was used to remove the noise. Data samples collected from each participant were then rescaled between 0 and 1 so they could be combined to fit in the classification models. The standard deviation of the data of 4 participants is shaded, and the biceps showed the largest standard deviation because it is involved in all workouts.

### 2.4. Classification of Targeted Muscles during 3 Workouts

After being normalized, data were labeled and fit into machine learning models. The whole dataset was annotated in 3 classes corresponding to 3 workouts. In this study, the authors considered three machine learning classification algorithms to classify the targeted muscles during each workout, i.e., Decision Tree (DT), Support Vector Machine (SVM), and k-Nearest Neighbors (KNN). The fine DT model has a simple structure that consists of nodes representing the attributes and other elements called branches and leaves, with maximum splits of 100. Besides the fine DT, fine Gaussian multiclass SVM was employed that uses the Gaussian kernel with kernel width set to the square root of the numbers of the predictors divided by 4. The last classifier used in this study is fine KNN, which is a basic classifier and makes finely detailed distinctions between classes with the number of neighbors set to 1. To ensure the stability of these models on the test data, K-Fold Cross Validation was employed during the training process of each model. In this method, the data are split into k subsets. In each subset, holdout method is applied so that a part of the training data is removed and used later to obtain predictions from the model trained on the rest of the data. So, in K-Fold Cross Validation, the holdout method is repeated k times. For each time, every subset is used as the test set or validation set, and the rest of the subsets are gathered to shape into the training set. All these models were run on MATLAB R2021b software, the number of subsets was 20, and 25% of total samples were set aside for test/validation data.

## 3. Results

### 3.1. Assessment of the Performance of Textile-Based Sensor

**Figure 8:**
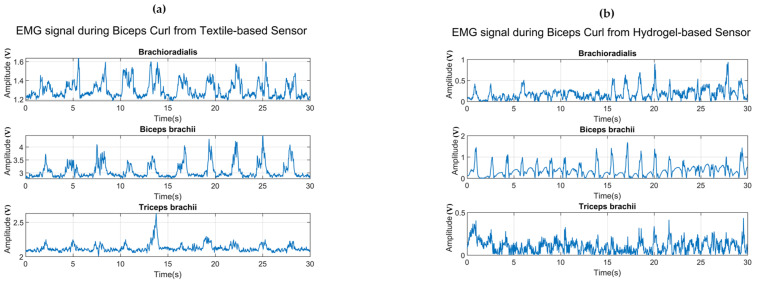
(**a**) EMG envelope data recorded by the textile-based sensor, (**b**) the hydrogel-based sensor.

To evaluate the performance of the proposed textile-based sensor, the authors compared EMG data collected by the hydrogel-based sensor and the proposed device. In this experiment, both types of electrodes were attached to the Myoware Muscle Sensor Board from SparkFun and used to record the EMG signal at two separate training sessions. The hydrogel-based electrode(Ag/AgCl 3M, Saint Paul, MN, USA) was used. The participant was asked to perform Biceps Curls for 30 s for each session with 1 min of rest between them to ensure that the muscles were entirely relaxing. Three channels of EMG signal corresponding to three targeted muscles are illustrated in Figure 8.

In the Biceps Curl workout, the biceps brachii is defined as the most activated muscle, which is visible from the amplitude of the EMG signal recorded from both sensors in each channel. Each pulse in the plot represented the contraction of three muscles during the workout. As can be seen from the figure, the textile-based sensor caught less external noise. Moreover, the difference in amplitude of the EMG envelope signal recorded by the textile-based sensor between the defined muscles and the other muscles is more noticeable compared to the one recorded by the hydrogel-based sensor. According to the data acquisition protocol, each participant was asked to perform sets of three workouts for 90 s, i.e., Wrist Curl for the first 30 s, Biceps Curl for the next 30 s, and Dumbbell Kickback for the last 30 s. As mentioned in the previous part, brachioradialis is involved the most during Wrist Curl; while biceps brachii and triceps brachii are mainly activated during Biceps Curl and Dumbbell Kickback, respectively.

**Figure 9:**
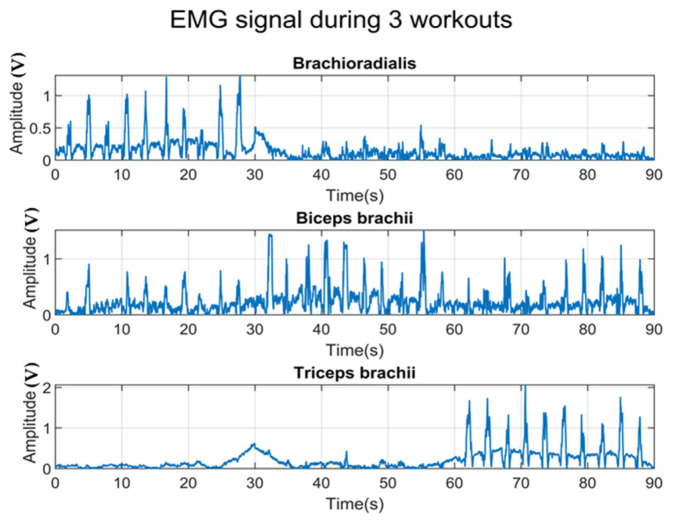
EMG data were collected from 3 muscles during 3 workouts. Each of them was carried out for 30 s (90 s in total) in the following order: Wrist Curl, Biceps Curl, and Dumbbell Kickback.

In Figure 9, each pulse represents the contraction of the muscle during the workout. It is noticeable that the EMG signal collected from the most activated muscle in each workout has a considerably high amplitude compared to the other two muscles.

Furthermore, this study aims to develop a wearable device to monitor EMG signals during a workout, so the experience of participants was considered as well. All participants stated that they felt comfortable with the textile-based electrodes. At the end of the data collection process, only one participant said they were uncomfortable because the armband, which was used to ensure better contact between the electrodes and the skin, caused the Myoware electrode connectors to leave marks on the skin.

### 3.2. Assessment of the Machine Learning Models

In this study, to evaluate textile-based sensor performance, simple ML models were used to classify the muscle groups that were primarily activated during each workout. To simplify the classification, the participants were asked to practice the workouts prior and ensure that during each specific workout, the targeted muscle was activated. The statistical metric used to evaluate the classification learner is accuracy [29]. In the classification task, the accuracy of a model indicates the percentage of correct predictions that it makes. In Binary Classification, the accuracy of a model is indicated as

(4) $$Accuracy=\frac{TP+TN}{TP+FP+TN+FN}\times 100\%$$

Because there are only two classes—positive and negative—in the above formula, TP, FP, TN, and FN are positive classes that are correctly predictive as positive, negative classes that are falsely predicted as positive, and negative classes that are correctly predicted as negative, and positive classes that are falsely predicted as negative, respectively, although the classification task in this study is a multi-class problem. So, accuracy, in this case, has the same definition as in the binary one, but is indicated in a more general formula:

(5) $$Multiclass\;Accuracy(y_{i},z_{i})=\frac{1}{n}\sum_{1}^{n}[[y_{i}==z_{i}]]$$

where n is the number of samples, [[…]] is the Iverson bracket that returns 1 when the expression within it is true, and 0 when it is false, y_i_ and z_i_ are the true and predicted output labels of the given data, respectively.

**Figure 10:**
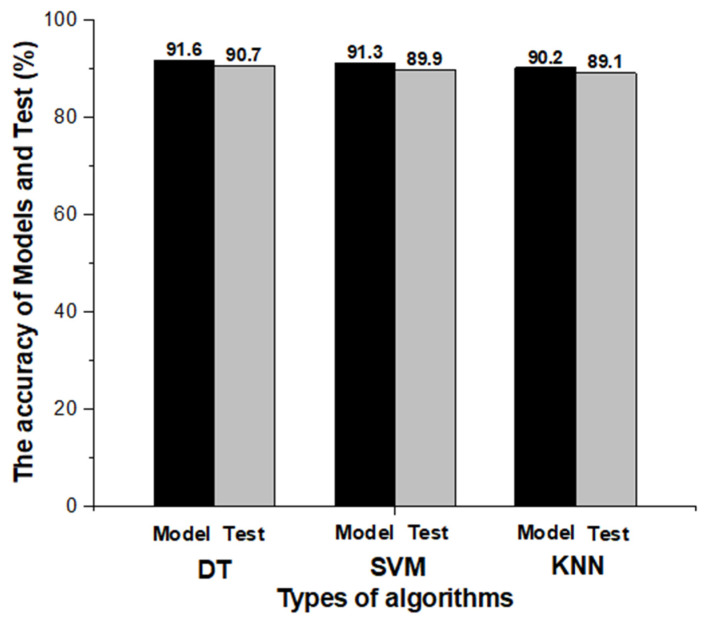
Comparison of the validation accuracy and test accuracy of three classification models used to classify three arm workouts.

**Figure 11:**
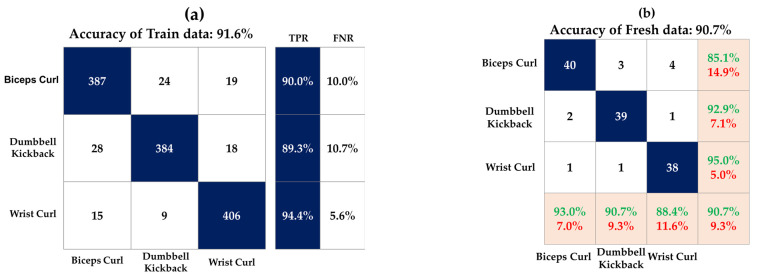
Confusion matrix of DT model on (**a**) train data and (**b**) test data.

**Figure 12:**
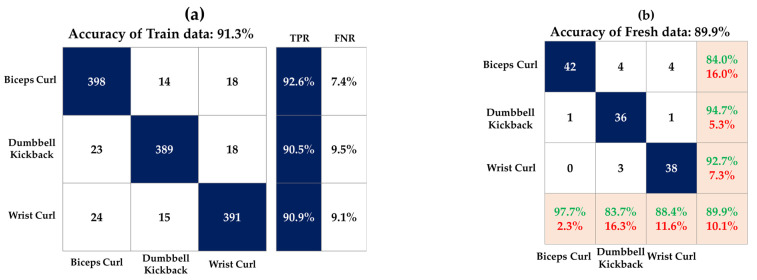
Confusion matrix of SVM model on (**a**) train data and (**b**) test data.

**Figure 13:**
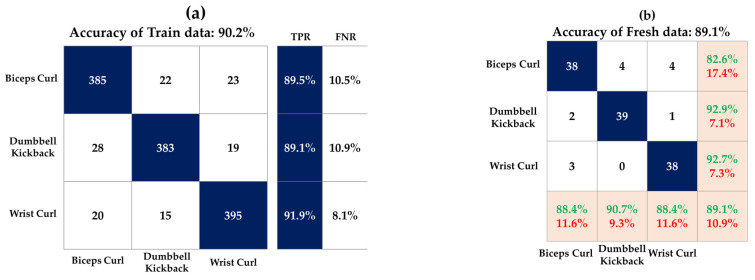
Confusion matrix of KNN model on (**a**) train data and (**b**) test data.

Figure 10 shows the accuracy of the model and fresh data for each DT, SVM, and KNN model. Figure 11, Figure 12 and Figure 13 show the confusion matrix of three classification models used in this study. This matrix visualizes the performance of a trained classification algorithm. Diagonal components represent a correct classification of a workout, and off-diagonal components represent the incorrect classification of a workout. The test data were the data of the participants and showed 90.7%, 89.9%, and 89.1% accuracy in the order of DT, SVM, and KNN, respectively. Although EMG envelope data were different for each participant, high accuracy was obtained because rescaling was performed so all the samples laid between 0 and 1. The reason for the error rate of 10% seems to be that it was difficult to classify the workout at the beginning of the workout, and it took time for the EMG signal to stabilize at the end of the workout. EMG data do not take long to stabilize, but this is because you work out continuously without a break.

**Figure 14:**
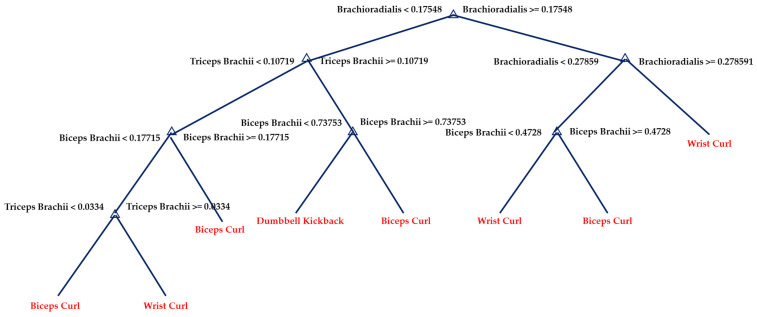
The graphical display of one tree in the DT model.

A Decision Tree is a kind of decision-making support tool that schematizes decision-making rules and their results in a tree structure(Figure 14).

## 4. Conclusions

Previous studies have focused on replacing hydrogel (wet electrodes) with dry electrodes. Other studies have focused on recognizing/classifying aerobic exercise, and motion, by analyzing EMG signals. In this study, high-purity SWCNTs were used to make neoprene into nine textile electrodes that could replace hydrogels and be fabricated in a wearable form. In addition, through LPF pre-processing, we obtained data that can be rescaled by removing noise of 5 Hz or higher. Each machine learning model was trained with the features of the EMG signal and tested with new data from the participants, showing a high workout classification accuracy of over 90%. After the COVID-19 pandemic, home training became popular as interest in exercise increased along with the growth of the healthcare market. This study aimed at developing a wearable device in the form of an arm sleeve using textile-based electrodes, and applied machine learning to classify arm workouts with EMG data recorded in the proposed device—a wearable device that can help you work out at the gym or home without a PT. The device can monitor what workouts the user is doing. Once a workout has been classified, these data have the potential to be informative, such as providing details about the exerciser’s posture, whether or not the target area was loaded, the number of workouts, and the appropriate amount of time for each workout. We proceeded with the arm, which is the basis of the workout, and in future studies, the authors consider developing an algorithm along with EMG data analysis, which can help determine whether the posture is correct. The purpose of the workout is to prevent injury and to ensure that the target area is well loaded. Through future research, it will be possible to design an algorithm that can determine whether the target area is well stimulated based on the workout classification. In addition, since the sleeve device can assess and monitor remotely, it can be used in all digital health spaces that require non-face-to-face contact.

## Figures and Tables

**Table 1:** Anthropometric information of the participants.

Participant	Age	Gender	Height (m)	Arm Length (cm) Shoulder–Wrist
1	28	Male	1.80	60
2	28	Male	1.82	63
3	28	Male	1.70	58
4	29	Male	1.78	69

## Data Availability

Not applicable.
